# Spatial Variation in COVID-19 Mortality in New York City and Its Association with Neighborhood Race, Ethnicity, and Nativity Status

**DOI:** 10.3390/ijerph20176702

**Published:** 2023-09-01

**Authors:** Samantha Friedman, Tabassum Z. Insaf, Temilayo Adeyeye, Jin-Wook Lee

**Affiliations:** 1 Department of Sociology, University at Albany, SUNY, 348 Arts & Sciences Building 1400 Washington Avenue, Albany, NY 12222, USA; 2 Bureau of Environmental and Occupational Epidemiology, New York State Department of Health, 1203 Corning Tower, Empire State Plaza, Albany, NY 12223, USA; tabassum.insaf@health.ny.gov (T.Z.I.); temilayo.adeyeye@health.ny.gov (T.A.); 3 Department of Epidemiology and Biostatistics, School of Public Health, University at Albany, SUNY, 1 University Place, Rensselaer, NY 12144, USA; 4 Department of Environmental Health Sciences, School of Public Health, University at Albany, SUNY, 1 University Place, Rensselaer, NY 12144, USA; 5 Center for Social and Demographic Analysis, University at Albany, SUNY, 321 University Administration Building, Albany, NY 12222, USA; jwlee@albany.edu

**Keywords:** COVID-19 mortality, minority health disparities, immigrant neighborhoods, New York City, spatial analysis

## Abstract

We examined the association between variation in COVID-19 deaths and spatial differences in the racial, ethnic, and nativity-status composition of New York City neighborhoods, which has received little scholarly attention. Using COVID-19 mortality data (through 31 May 2021) and socioeconomic and demographic data from the American Community Survey at the Zip Code Tabulation Area level as well as United-Hospital-Fund-level neighborhood data from the Community Health Survey of the New York City Department of Health and Mental Hygiene, we employed multivariable Poisson generalized estimating equation models and assessed the association between COVID-19 mortality, racial/ethnic/nativity-status composition, and other ecological factors. Our results showed an association between neighborhood-level racial and ethnic composition and COVID-19 mortality rates that is contingent upon the neighborhood-level nativity-status composition. After multivariable adjustment, ZCTAs with large shares of native-born Blacks and foreign-born Hispanics and Asians were more likely to have higher COVID-19 mortality rates than areas with large shares of native-born Whites. Areas with more older adults and essential workers, higher levels of household crowding, and population with diabetes were also at high risk. Small-area analyses of COVID-19 mortality can inform health policy responses to neighborhood inequalities on the basis of race, ethnicity, and immigration status.

## 1. Introduction

New York City (NYC) bore the most significant share of the brunt of the COVID-19 pandemic within the United States [1,2]. Four of the five counties that make up NYC—Bronx, Kings, New York, and Queens—had numbers of deaths from COVID-19 in the top 20 counties out of the more than 3000 counties within the United States. At the time of this writing, about 1 in 24 deaths from COVID-19 in the U.S. were in NYC, far exceeding the share of the U.S. population living in NYC—1 in 40 people in the U.S. lived in NYC [3].

Racial and ethnic disparities in COVID mortality have been a prominent, negative outcome of the pandemic. Blacks and Hispanics were more likely to die from COVID-19 than Whites, and the percentages of Blacks and Hispanics dying from COVID-19 exceeded their shares of the population, respectively [4,5]. In New York State (NYS), the in-hospital fatality rates for Blacks and Hispanics that died from COVID-19 were 5.38 and 3.48 times higher than the fatality rate for non-Hispanic Whites, respectively [6]. Research of a cohort study of patients in NYC found that although Black and Hispanic patients were more likely to test positive for COVID-19, once they were hospitalized, Black patients were significantly less likely to die than White patients; Hispanic patients’ risk of mortality was no different than that of Whites, suggesting that a disproportionate share of the mortality of Blacks and Hispanics occurred outside of the hospital at their homes and in their neighborhoods [7].

There have been several studies that examined racial and ethnic disparities in COVID-19 mortality at the neighborhood level in NYC [8,9,10,11,12,13,14,15,16,17,18]. Descriptive evidence revealed that neighborhoods with people of color, like East New York, East Harlem, and Corona, had disproportionately higher rates of COVID-19 mortality than predominantly White neighborhoods such as the Upper West Side and Greenwich Village [10,13,18]. However, multivariable analyses revealed mixed findings with respect to the association between the racial and ethnic composition of neighborhoods and COVID-19 mortality rates. For example, some studies found that COVID-19 mortality did not significantly vary by the racial and ethnic composition of neighborhoods, when controlling for neighborhood socioeconomic status and the share of essential workers [8,11,18]. Other research, however, found a significant association between racial and ethnic composition and neighborhood-level COVID-19 mortality rates [12,15,17,18,19]. Among the latter studies, there were differences in the nature of the associations. For example, two studies found no significant association between neighborhood Asian composition and COVID-19 mortality [12,17]. Other studies just focused on White and Hispanic composition and did not examine Black and Asian composition, making it difficult to know whether neighborhoods of particular minority composition had higher rates of COVID-19 mortality [15,18,19].

The racial and ethnic composition of NYC neighborhoods is complicated by the fact that NYC has been a prominent destination for immigrants. In 2017, 37.1% of NYC’s population was born outside the United States, and sizeable shares of the White, Black, Hispanic, and Asian populations—22.0%, 32.3%, 40.3%, and 70.9%, respectively,—were immigrants [20]. It is possible that the differences in the association between neighborhood-level racial and ethnic composition and COVID-19 mortality rates found in recent studies were attributable to the variation in the shares of foreign-born population within each racial and ethnic group in NYC neighborhoods. However, only one study examined racial and ethnic composition of NYC neighborhoods by nativity status and its effect on COVID-19 mortality rates, but that study was limited in the time period examined and did not control for community-level health indicators [16].

This study sought to build on this scant research and systematically explored the association between spatial variation in COVID-19 mortality rates across neighborhoods in NYC, defined at the ZIP Code Tabulation Area (ZCTA) level, as it relates to the neighborhood-level racial, ethnic, and nativity status composition, socioeconomic, demographic, and health characteristics. Given that racial-ethnic-nativity-status-specific mortality rates were unavailable by neighborhoods in NYC, this study provided a way to explore the association between COVID-19 mortality and the racial, ethnic, and nativity-status composition of neighborhoods in NYC to identify areas that were hardest hit by the pandemic. Moreover, because many areas in NYC were composed of large shares of immigrant populations that are Hispanic and Asian, this study offered the opportunity to build on existing research that has treated race and ethnicity independent of nativity status.

## 2. Materials and Methods

We used a cross-sectional approach to conduct a spatial epidemiological analysis of COVID mortality among all residents of NYC from 29 February 2020 to 31 May 2021. The primary outcome of interest was the COVID-19 mortality rate for each ZCTA, calculated as the total number of COVID-19 deaths in a ZCTA per 100,000 population. Data on COVID-19 deaths at the ZCTA level were acquired from data released daily by the New York City Department of Health and Mental Hygiene (NYC DOHMH) [21]. We used COVID-19 mortality rates as our outcome rather than COVID-19 infection rates because the former was the most significant endpoint in the pandemic. Moreover, data on COVID-19 infection rates were less valid as an outcome measure of the pandemic. Infection rates could have been higher in a neighborhood because there were more actual COVID-19 cases or because there were more tests available. This was of particular concern in areas with greater shares of non-White or minority populations. In those areas, there was probably a lower rate of infection because of a lack of tests rather than because of an actual lower rate of infection.

ZCTAs are generalized areal representations of United States Postal Service (USPS) ZIP Code service areas. Data on demographic and socioeconomic characteristics of ZCTA areas, including the racial, ethnic, and nativity-status composition; age composition; and concentrated disadvantage index, were obtained from the 2014–2018, 5-year release of the American Community Survey (ACS) available via the IPUMS NHGIS website maintained by the University of Minnesota Population Center [22]. Data on community health indicators were acquired from restricted data from the 2016–2018 New York Community Health Survey (CHS), a telephone survey conducted annually by the NYC DOHMH [23].

**Figure A1:**
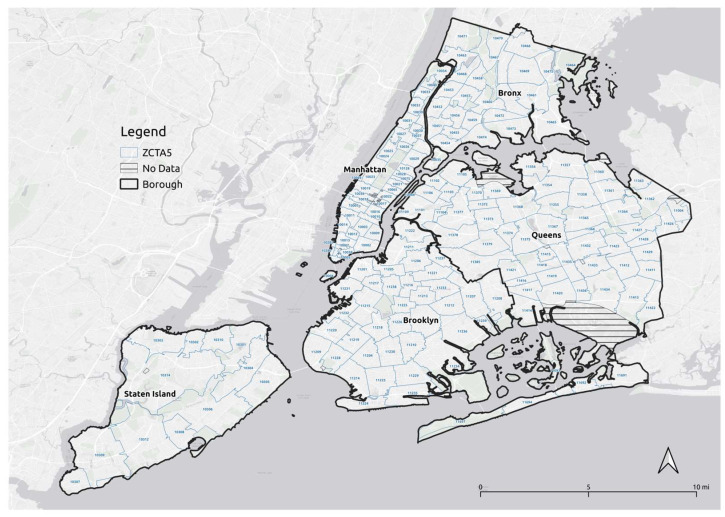
Neighborhood Map of ZCTAs New York City.

Our primary unit of analysis was at the neighborhood level that is defined by ZCTAs. Appendix A Figure A1 provided a map of the neighborhoods across NYC that were included in our analysis, and Appendix A Table A1 showed the names of the neighborhoods on the map. We linked mortality and sociodemographic data for 177 ZCTAs in NYC. Our key variables of interest focused on the race, ethnicity, and nativity-status of the population in the ZCTAs. In the ACS, race, ethnicity, and nativity-status were self-defined by respondents [24]. The question on race asked persons in households to identify their race—“What is Person 1’s race?” and respondents marked an X next to the boxes they desired from the following socially constructed choices of race—White, Black, American Indian or Alaska Native, several categories of Asian, and some other race [24]. The question we used for ethnicity asked persons in the household to identify whether they were of Hispanic, Latino, or Spanish origin; respondents made one of the following choices—no they were not of Hispanic, Latino, or Spanish origin; or that they were of Hispanic, Latino, and Spanish origin and then selected the specific category (i.e., Mexican, Puerto Rican, Cuban, or filled in “another Hispanic, Latino, or Spanish origin”) [24]. Nativity status was based upon the question posed to each person in the household that asked, “Where was this person born?”; respondents that chose the U.S. had to print the name of the state; those that chose outside the U.S. had to specify the country in which they were born. Nativity status referred to native- and foreign-born segments of each racial and ethnic group. Foreign-born status was defined as individuals born outside of the U.S., excluding Puerto Rico and other U.S. islands, that were born to parents who were not U.S. citizens; native-born status composed the residual group.

We used the 2014–2015 ACS Summary File (SF) that provided tables of aggregated individual-level data at various levels of geography. For the purposes of our study, we used tables that combined the race, ethnicity, and nativity status of respondents at the ZCTA level of analysis. We measured the racial, ethnic, and nativity-status composition with the following variables: the percentages of native- and foreign-born Blacks, Hispanics, and Asians and the percentage of foreign-born, non-Hispanic Whites, with the percentage of native-born, non-Hispanic Whites (hereafter referred to as native-born Whites) as the reference group. It should be noted that for Blacks and Asians, the tables provided in the ACS SF included both Hispanics and non-Hispanics. The ACS only provided data stratified by Hispanic origin for Whites. Hispanics included those of all races. Therefore, there was some overlap between our categories of Black and Asian with the category of Hispanic. However, according to the 2014–2018 ACS data for NYC, only 9.6% of Blacks and 0.9% of Asians identified that they were of Hispanic origin; and among Hispanics, 8% and 0.4% identified as Black and Asian, respectively [25]. Thus, the overlap was minimal. Our categorization of racial, ethnic, and nativity-status composition was consistent with other research [13].

To gauge the level of disadvantage of communities, we created a concentrated disadvantage index (CDI) [26]. The index was based upon five variables—poverty level, unemployment rate, welfare receipt, percent of female-headed households, and percent of children under 18 years of age in each ZCTA. We conducted a principal component analysis, which confirmed that there was a single factor onto which these factors loaded. Then we created z-scores and added the measures into a single index. High scores on this index indicated high levels of concentrated disadvantage. Since a z score can range from −3 to +3 for each measure, the plausible values of the CDI were from −15 to +15 for a given census tract. We also created a variable measuring the percentage of those aged 65 and older. We included a measure of the percentage of housing units in which there were more than one person per room to indicate household crowding at the neighborhood level. To gauge the percentage of essential workers in the neighborhood, we followed the methodology of Glaeser and colleagues [27].

We measured the health of communities using data for 42 University Hospital Fund (UHF) areas from the CHS. To assign ZCTAs to UHFs, we used a population-weighted centroid methodology on the basis of where the majority of the population fell [28,29]. To gauge the health status of the population across NYC neighborhoods, we included the percentage of the population in UHF areas that were told that they have: (1) high blood pressure; and (2) diabetes. We included these variables because in NYC, hypertension and diabetes have been found to be among the most common comorbidities of COVID-19 hospitalization [30] and mortality [31]. Moreover, neighborhoods that are disproportionately Black and Hispanic have higher levels of hypertension and diabetes than White and Asian neighborhoods, thereby making these neighborhoods more vulnerable to COVID-19 mortality [32]. Finally, we included an indicator variable indicating whether the ZCTA included any area that had been historically redlined or was graded a “D” grade by the Home Owner’s Loan Corporation (HOLC).

We used Poisson generalized estimating equation (GEE) models, with the log of the total population as the offset to estimate Mortality Incidence Rate Ratios (MIRR) associated with each risk factor. The MIRR was used to assess whether mortality rates were elevated in ZCTAS with a higher proportion of individuals with a specific risk factor. Using Poisson regression, the MIRR was calculated as the exponential of the regression coefficient for each independent variable. The MIRR was interpreted as the risk ratio of the COVID-19 mortality rate increase associated with a one-percentage point unit increase in a particular independent variable, while holding other variables constant. We further estimated predicted risk and residuals from our final model to assess the spatial distribution of model estimates. A Moran’s I test for spatial autocorrelation of COVID-19 mortality indicated that there was significant spatial autocorrelation. The GEE model allowed adjustment for the geographical clustering of ZCTAs.

Furthermore, we used a nested model approach to evaluate the association across multiple risk factors. First, we estimated models that only included the racial, ethnic, and nativity-status composition variables. Then, we adjusted the estimates of compositional variables for relevant sociodemographic characteristics. Then, we evaluated the association between health characteristics of neighborhoods by including additional variables for the prevalence of hypertension and diabetes. Finally, we included a model with our redlining indicator variable. The model including hypertension showed the effect of that health variable was non-significant, so the final results are presented only with the model including diabetes. Our analyses were performed using the GEE procedure in SAS™ statistical software Version 9.4. (SAS Institute Inc., Cary, NC, USA).

## 3. Results

### 3.1. Descriptive Results

Table 1 provides descriptive statistics for the dependent and independent variables that were used in our multivariate analysis. The mean COVID-19 mortality rate across the 177 ZCTAs was 94.57 per 100,000 population (Range 0–390). The mean% of native-born Whites was 28.66%, which was the largest average among the compositional variables, and there was a lot of variability (SD: 22.12) in its distribution across ZCTAs, with the minimum and maximum values ranging from 0.56% to 89.66%. The mean% distributions of native-born Blacks and Hispanics were 15.02% and 15.84%, respectively; and both variables had a large range of values across ZCTAs, as their standard deviations were 16.52 and 11.06, respectively. Foreign-born Hispanics and Asians had mean% distributions of 10.21% (Range 0–46.3) and 10.37% (Range 0.08–58.44), respectively. The percentage distribution of foreign-born Whites and Blacks and native-born Asians were among the lowest mean percentages across ZCTAs at 7.54%, 6.71%, and 4.51%, respectively.

Table 1 shows variability across ZCTAs for our socioeconomic, demographic, health, and institutional discrimination variables. The average CDI was 0, ranging from a low of −7.62 to a high of 11.45. The mean% aged 65 and older was 14.30% with a range from 0.46% to 28.98%. The percentage distribution of essential workers, however, showed less variation than the other characteristics. The average% essential workers was 71.39% with a standard deviation of only 1.84 units. The mean% of crowded housing units was 8.30, ranging from 0.94 to as high as 29.65. The average% distributions of those told by a doctor that they have diabetes and high blood pressure were 10.66% (Range 3.86–17.15) and 26.52% (Range 15.83–37.95), respectively. The proportion of ZCTAs that contained formerly redlined areas was 0.70.

### 3.2. Multivariable Results

What was the association between racial, ethnic, and nativity composition and COVID-19 mortality across ZCTAs? In Table 2 in the unadjusted analysis (Model 1), for every unit increase in percentage of foreign-born Whites in any ZCTA, we observed a 2.7% increase in the COVID-19 mortality rate (MIRR = 1.027; 95% CI 1.018, 1.035) (Table 2). Similar associations were seen with increases in the percentages of native-born Blacks (MIRR = 1.010; 95% CI 1.003, 1.016), foreign-born Blacks (MIRR = 1.018; 95% CI 1.008, 1.027), native-born Hispanics (IRR = 1.010; 95% CI 1.002, 1.019), foreign-born Hispanics (MIRR = 1.015; 95% CI 1.008, 1.023), and foreign-born Asians (MIRR = 1.038; 95% CI 1.020, 1.056). However, an increase in the percentage of native-born Asians was significantly associated with a decrease in COVID-19 mortality (MIRR = 0.934; 95% CI 0.887, 0.984).

Adjusting for the CDI and percentages of those aged 65 and older, essential workers, and of crowded housing units, the aforementioned associations persisted only for the covariates for percentages: native-born Black, foreign-born Hispanic, and foreign-born Asian. Older age composition, percentage essential workers, and household crowding were significantly associated with an increased risk of COVID-19 mortality (Table 2, Model 2). A unit increase in the percentage of the population aged 65 and older was associated with a 4.7% increase in the COVID-19 mortality rate (MIRR = 1.047; 95% CI 1.034, 1.061). A one-unit increase in the percentage essential workers was associated with a 6.8% increase in the COVID-19 mortality rate (MIRR = 1.068; 95% CI 1.024, 1.114), and a one-unit increase in the percentage of crowded housing units was associated with a 2.3% increase in the COVID-19 mortality rate (MIRR = 1.023; 95% CI 1.010, 1.037).

Research suggests a link between diabetes and COVID-19 mortality [33,34]. Therefore, we further adjusted for the percentage of people told by doctors that they have diabetes per ZCTA in Model 3 and observed that a one-unit increase in the percentage of people with diabetes was associated with a 1.8% increase in the COVID-19 mortality rate, controlling for other factors, although the coefficient was not statistically significant (MIRR 1.18; 95% CI 0.996, 1.040). Additional adjustment for health status within each ZCTA did not change the established associations in Model 2, except for the coefficient for native-born Asians, which became statistically significant.

**Figure A2:**
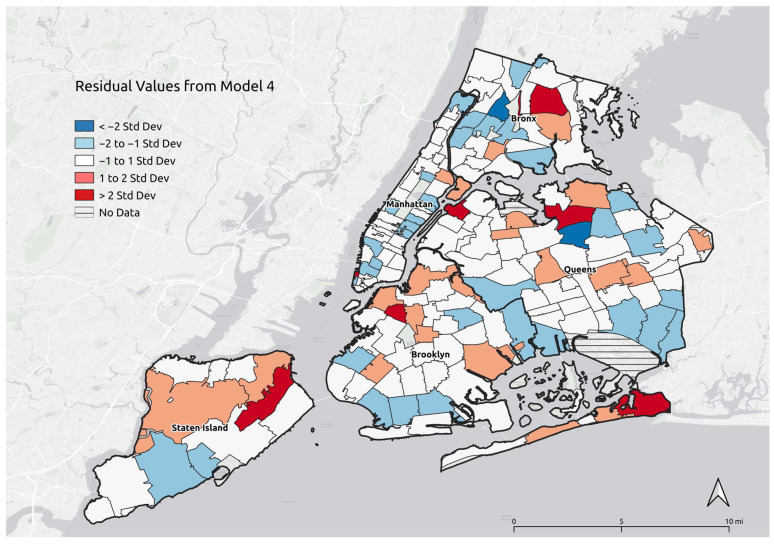
Map of residuals from multivariate model of racial, ethnic, and nativity-status disparities in New York City, 29 February 2020 to 31 May 2021.

In Model 4, we adjusted for the presence of redlining in the ZCTA because research has suggested a link between redlining and COVID-19 infection in NYC [11]. However, the coefficient for redlining was not statistically significant. Most of the established associations present in Model 3 remained the same. However, the coefficient for native-born Asian became non-significant. In contrast, the coefficient for diabetes became statistically significant. The results in Model 4 show that a one-unit increase in the percentage of people with diabetes was associated with a 2.4% increase in the COVID-19 mortality rate, controlling for other factors, although the coefficient was not statistically significant (MIRR 1.024; 95% CI 1.001, 1.048). The spatial distribution of the residual error terms from our final spatial error model (Model 4) showed that our model fit is equivalent in neighborhoods across NYC (see Appendix A Figure A2).

**Figure 1:**
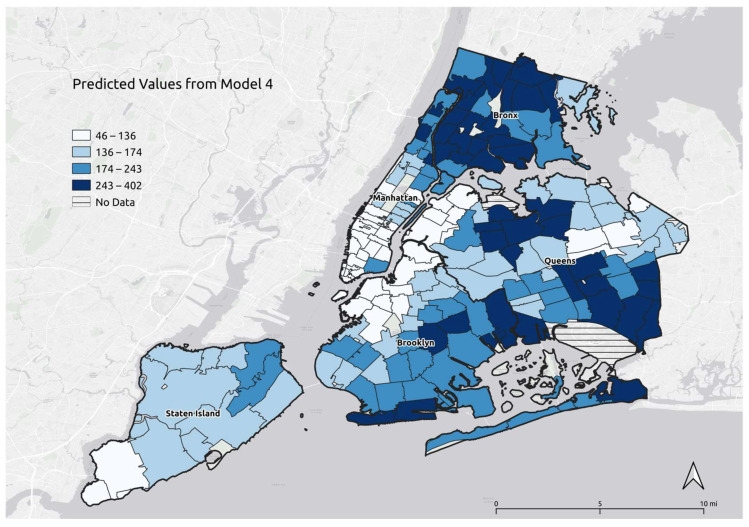
Predicted rates for COVID-19 mortality per 100,000 population at the ZCTA level in New York City, 29 February 2020 to 31 May 2021.

Figure 1 reports the predicted COVID-19 mortality rates per 100,000 population at the ZCTA level. These maps used the predicted rates of COVID-19 mortality and residuals based on Model 4, which included all the social determinants used in the study. The predicted mortality provided a smoothed map, which may be used in conjunction with the observed values, to show the areas that experienced the greatest levels of mortality. In connection with previous research [13], the maps suggest that the highest predicted values were in areas with higher percentages of native-born Blacks and foreign-born Hispanics and Asians (Figure 1), suggesting that the variables in the model predicted COVID-19 mortality within two standard deviations of the observed values. Out of 177 ZCTAs, only six areas had observed values that were significantly higher than the model predictions (Appendix A Figure A2—see dark red values on the map), suggesting that there may be additional unmeasured factors that could have contributed to higher mortality in these areas. There were only two areas where observed values were lower than predicted values.

## 4. Discussion

The main objective of this study was to examine the association between spatial variation in COVID-19 mortality rates across neighborhoods in NYC, as it relates the racial, ethnic, and nativity status composition of the population in those neighborhoods. We sought to identify neighborhoods of color and immigrants in NYC that have been hardest hit by the pandemic. We also examined what factors were associated with higher rates of COVID-19 mortality in neighborhoods. Our analyses revealed that spatial variation in COVID-19 mortality rates was not just a function of racial and ethnic composition of neighborhoods in NYC as previous research has shown [8,9,10,11,12,13,14,15,17,18]. It was also highly dependent upon the nativity-status composition of neighborhoods, supporting the results of one study in the literature [16] and suggesting that future research should consider all three of these characteristics of neighborhoods in examining spatial variation in COVID-19 mortality.

Our multivariate analyses revealed that there are several important characteristics of neighborhoods, aside from the racial, ethnic, and nativity-status composition that related to the spatial distribution of COVID-19 mortality rates across ZCTAs in NYC. The percentages of the population: aged 65 and over, that were essential workers, living in crowded housing units; and the percentage of the population in UHFs with diabetes were all positively and significantly related to the level of COVID-19 mortality in neighborhoods across NYC.

It is established that older persons have a higher mortality rate from COVID-19 than younger persons [35,36]. The age composition of the population in neighborhoods was also a likely key factor explaining the association between the spatial variation of COVID-19 deaths per 100,000 population and percentages of the population that are foreign-born White and native-born Asian. ZCTAs with larger percentages of foreign-born Whites tended to be older, which in turn led to higher neighborhood-averaged mortality rates. There was a larger proportion of younger people in ZCTAs with native-born Asians. Data for NYC as a whole revealed that 50% of native-born Asians were under 18 years old, relative to only 4% of foreign-born Asians [37]. The loss of statistical significance of the association between COVID mortality and percentage native-born Asians after controlling for ZCTA age composition was consistent with this demographic pattern.

Many of the ZCTAs that had large shares of native-born Black population and greater levels of COVID-19 mortality rates were in areas with high levels of diabetes, which has been shown to be a reflection of the persistent racial segregation faced by Blacks in NYC [38,39]. Neighborhoods plagued by segregation had higher levels of crime and greater levels of disinvestment that result in poorer structural resources like a lack of high-quality healthcare and educational institutions and an absence of recreational facilities and first-rate supermarkets [40]. Neighborhoods with large shares of native-born Blacks in NYC were more likely to have poorer health outcomes and higher levels of mortality than neighborhoods with greater shares of other minority groups because Black–White residential segregation has been consistently in the highest range for five decades, exceeding the segregation of other groups from Whites and setting NYC apart from many other cities in the U.S. [38,39,41,42].

Our analysis showed that neighborhoods with large shares of foreign-born Hispanics and Asians were particularly vulnerable to COVID-19 mortality, even after controlling for neighborhood-level age composition, socioeconomic status, demographic factors, the health of residents, and redlining in these areas. Therefore, our results suggested that there were other factors likely correlated with the variation in COVID-19 deaths per 100,000 population in NYC. Because many of the deaths in NYC resulted from the population becoming ill at the outset of the pandemic, when masks were not mandated and stay-at-home orders were not in place, we suspect that neighborhoods of Hispanic and Asian immigrants were likely to be more vulnerable because of contact with others who recently traveled from overseas. In addition, immigrant neighborhoods tended to have extensive co-ethnic social networks, particularly in the form of friendship and kinship ties, which likely created greater levels of exposure to COVID-19 [43]. Moreover, NYC levels of residential segregation of Hispanics and Asians from Whites were unusually high, suggesting that immigrants cluster in neighborhoods with co-ethnics, raising their vulnerability to COVID-19 [44,45].

Our predicted rate map provided a visual representation of the variability in rates of COVID-19 mortality as predicted by social determinants considered in this study. The rates were highest in ZCTAs where the native-born Black population was very large, including Kingsbridge-Riverdale, High Bridge-Morrisania, Pelham-Throgs Neck, East and Central Harlem, East Flatbush-Flatbush, Bedford Stuyvesant-Crown Heights, and Jamaica [13]. The predicted COVID-19 mortality rates were also high in ZCTAs with large shares of foreign-born Hispanic population, including West Queens, East Harlem, and Jamaica, and in ZCTAs with large shares of foreign-born Asian population, including Flushing-Clearview, Ridgewood-Forest Hills, and Southwest Queens [13].

Our study has some limitations. Although the ACS data were collected at the census block-group level, based on the underlying population distribution at that level of analysis, we were limited to conducting the analysis at the ZCTA-level because the COVID mortality data were only available at the zip-code level. In addition, the mortality data were not released by race, ethnicity, or nativity status. Therefore, we could not examine correlates of COVID-19 mortality for specific groups. Finally, the data were released at the ZCTA level, which aligns with postal service distribution areas rather than neighborhoods as defined by the residents of NYC.

## 5. Conclusions

Our analyses made clear that differences in COVID-19 mortality by the racial, ethnic, and nativity-status composition of neighborhoods reflected spatial inequalities that existed long before the pandemic. NYC is one of the most racially and ethnically segregated cities in the US. Decades of racial and ethnic residential segregation and disinvestment and the resultant poverty and unemployment have tragically ended many lives in neighborhoods of color and immigrant neighborhoods. Investment in the infrastructure of these neighborhoods is needed so that future lives are not lost.

## Figures and Tables

**Table 1:** Descriptive Statistics for Dependent and Independent Variables.

Variable	Mean	Standard Deviation	Min	Max
*COVID-19 Mortality Rate*	94.57	75.12	0	390
*Racial/Ethnic/Nativity Status Composition*				
Percentage:				
Native-born White	28.66	22.12	0.56	89.66
Foreign-born White	7.54	6.82	0.11	47.52
Native-born Black	15.02	16.52	0.14	62.81
Foreign-born Black	6.71	9.47	0	46.99
Native-born Hispanic	15.84	11.06	1.12	46.66
Foreign-born Hispanic	10.21	9.81	0	46.3
Native-born Asian	4.51	4.06	0	16.13
Foreign-born Asian	10.37	10.30	0.08	58.44
*Socioeconomic and Demographic Variables*				
Concentrated disadvantage index (CDI)	0	4.36	−7.62	11.45
Percentage aged 65 and older	14.30	5.04	0.46	28.98
Percentage essential workers	71.39	1.84	67.11	75.55
Percentage of crowded housing units	8.30	4.89	0.94	29.65
*Health Status of the Community*				
Percentage told they have diabetes	10.66	3.96	3.86	17.15
Percentage told they have high blood pressure	26.52	6.40	15.83	37.95
*Institutional Discrimination*				
ZCTA contained redlined area (1 = yes, 0 = no)	0.70	0.46	0	1
N	177			

NOTE: All values were percentages except for the CDI which was a sum of z-scores and the redlining indicator, which was a value of 1 if there was a redlined area in the ZCTA and 0 if there was not.

**Table 2:** Poisson GEE Models of COVID-19 Deaths, New York City, 29 February 2021 to 31 May 2021.

	Model 1	Model 2	Model 3	Model 4
Variable	MIRR (95% CI)	MIRR (95% CI)	MIRR (95% CI)	MIRR (95% CI)
Percentage:				
Foreign-born White	1.027 ***	1.006	1.004	1.002
	(1.018, 1.035)	(0.998, 1.015)	(0.995, 1.013)	(0.993, 1.011)
Native-born Black	1.010 **	1.010 ***	1.009 ***	1.008 **
	(1.003, 1.016)	(1.005, 1.016)	(1.004, 1.015)	(1.002, 1.014)
Foreign-born Black	1.018 ***	1.002	0.999	1.000
	(1.008, 1.027)	(0.991, 1.013)	(0.988, 1.011)	(0.988, 1.011)
Native-born Hispanic	1.010 *	1.005	1.002	1.002
	(1.002, 1.019)	(0.996, 1.015)	(0.992, 1.013)	(0.992, 1.012)
Foreign-born Hispanic	1.015 ***	1.012 ***	1.011 ***	1.012 **
	(1.008, 1.023)	(1.003, 1.020)	(1.002, 1.019)	(1.004, 1.019)
Native-born Asian	0.934 *	0.949	0.944 *	0.950
	(0.887, 0.984)	(0.899, 1.002)	(0.896, 0.994)	(0.900, 1.003)
Foreign-born Asian	1.038 ***	1.024 *	1.024 *	1.021 *
	(1.020, 1.056)	(1.004, 1.044)	(1.005, 1.044)	(1.001, 1.042)
Concentrated disadvantage index		1.010	1.007	1.007
		(0.989, 1.033)	(0.985, 1.029)	(0.986, 1.029)
Percentage aged 65 and older		1.047 ***	1.047 ***	1.051 ***
		(1.034, 1.061)	(1.034, 1.060)	(1.036, 1.066)
Percentage essential workers		1.068 **	1.057 *	1.055 *
		(1.024, 1.114)	(1.010, 1.105)	(1.010, 1.103)
Percentage of crowded housing units		1.023 ***	1.025 ***	1.023 ***
		(1.010, 1.037)	(1.011, 1.038)	(1.011, 1.037)
Percentage told have diabetes			1.018	1.024 *
			(0.996, 1.040)	(1.001, 1.048)
ZCTA contained redlined area				1.122
				(0.998, 1.261)
Constant	0.001 ***	0.000 ***	0.00001 ***	0.00001 ***
	(0.0006, 0.001)	(0.0000, 0.0000)	(0.0000, 0.0002)	(0.0000, 0.0002)
QIC	−10,787.216	−14,703.009	−14,947.470	−15,223.392
N	177	177	177	177

* *p* ≤ 0.05; ** *p* ≤ 0.01; *** *p* ≤ 0.001. MIRR = Mortality Incidence Rate Ratio. CI = Confidence Interval.

## Data Availability

The COVID-19 data presented in this study are available at: https://github.com/nychealth/coronavirus-data/tree/master/totals (accessed on 1 June 2021). The socioeconomic and demographic data from the 2014–2018, 5-year release of the ACS are available at: https://data2.nhgis.org/main# (accessed on 10 September 2020). Data on community health indicators were acquired from restricted data from the 2016–2018 New York Community Health Survey (CHS), a telephone survey conducted annually by the NYC DOHMH. Restrictions apply to the availability of these data; permission must be obtained from the NYC DOHMH; the process may be started here—https://nycdohmh.surveymonkey.com/r/EpiDataForm (accessed on 10 September 2020).

## Appendix A

**Table A1:** ZCTAs in New York City by Borough and Neighborhood Names.

ZCTA	Borough	Neighborhood Name	ZCTA	Borough	Neighborhood Name
10001	Manhattan	Chelsea-Clinton	11204	Brooklyn	Borough Park
10002	Manhattan	Union Square-Lower East Side	11205	Brooklyn	Downtown-Heights-Park Slope
10003	Manhattan	Union Square-Lower East Side	11206	Brooklyn	Williamsburg-Bushwick
10004	Manhattan	Lower Manhattan	11207	Brooklyn	East New York
10005	Manhattan	Lower Manhattan	11208	Brooklyn	East New York
10006	Manhattan	Lower Manhattan	11209	Brooklyn	Bensonhurst-Bay Ridge
10007	Manhattan	Lower Manhattan	11210	Brooklyn	East Flatbush-Flatbush
10009	Manhattan	Union Square-Lower East Side	11211	Brooklyn	Greenpoint
10010	Manhattan	Gramercy Park-Murray Hill	11212	Brooklyn	Bedford Stuyvesant-Crown Heights
10011	Manhattan	Chelsea-Clinton	11213	Brooklyn	Bedford Stuyvesant-Crown Heights
10012	Manhattan	Greenwich Village-SoHo	11214	Brooklyn	Bensonhurst-Bay Ridge
10013	Manhattan	Greenwich Village-SoHo	11215	Brooklyn	Downtown-Heights-Park Slope
10014	Manhattan	Greenwich Village-SoHo	11216	Brooklyn	Bedford Stuyvesant-Crown Heights
10016	Manhattan	Gramercy Park-Murray Hill	11217	Brooklyn	Downtown-Heights-Park Slope
10017	Manhattan	Gramercy Park-Murray Hill	11218	Brooklyn	Borough Park
10018	Manhattan	Chelsea-Clinton	11219	Brooklyn	Borough Park
10019	Manhattan	Chelsea-Clinton	11220	Brooklyn	Sunset Park
10020	Manhattan	Chelsea-Clinton	11221	Brooklyn	Williamsburg-Bushwick
10021	Manhattan	Upper East Side	11222	Brooklyn	Greenpoint
10022	Manhattan	Gramercy Park-Murray Hill	11223	Brooklyn	Coney Island-Sheepshead Bay
10023	Manhattan	Upper West Side	11224	Brooklyn	Coney Island-Sheepshead Bay
10024	Manhattan	Upper West Side	11225	Brooklyn	East Flatbush-Flatbush
10025	Manhattan	Upper West Side	11226	Brooklyn	East Flatbush-Flatbush
10026	Manhattan	Central Harlem-Morningside Heights	11228	Brooklyn	Bensonhurst-Bay Ridge
10027	Manhattan	Central Harlem-Morningside Heights	11229	Brooklyn	Coney Island-Sheepshead Bay
10028	Manhattan	Upper East Side	11230	Brooklyn	Borough Park
10029	Manhattan	East Harlem	11231	Brooklyn	Downtown-Heights-Park Slope
10030	Manhattan	Central Harlem-Morningside Heights	11232	Brooklyn	Sunset Park
10031	Manhattan	Washington Heights-Inwood	11233	Brooklyn	Bedford Stuyvesant-Crown Heights
10032	Manhattan	Washington Heights-Inwood	11234	Brooklyn	Canarsie-Flatlands
10033	Manhattan	Washington Heights-Inwood	11235	Brooklyn	Coney Island-Sheepshead Bay
10034	Manhattan	Washington Heights-Inwood	11236	Brooklyn	Canarsie-Flatlands
10035	Manhattan	East Harlem	11237	Brooklyn	Williamsburg-Bushwick
10036	Manhattan	Chelsea-Clinton	11238	Brooklyn	Bedford Stuyvesant-Crown Heights
10037	Manhattan	Central Harlem-Morningside Heights	11239	Brooklyn	Canarsie-Flatlands
10038	Manhattan	Lower Manhattan	11354	Queens	Flushing-Clearview
10039	Manhattan	Central Harlem-Morningside Heights	11355	Queens	Flushing-Clearview
10040	Manhattan	Washington Heights-Inwood	11356	Queens	Flushing-Clearview
10044	Manhattan	Upper East Side	11357	Queens	Flushing-Clearview
10128	Manhattan	Upper East Side	11358	Queens	Flushing-Clearview
10280	Manhattan	Lower Manhattan	11359	Queens	Flushing-Clearview
10301	Staten Island	Stapleton-St. George	11360	Queens	Flushing-Clearview
10302	Staten Island	Port Richmond	11361	Queens	Bayside-Little Neck
10303	Staten Island	Port Richmond	11362	Queens	Bayside-Little Neck
10304	Staten Island	Stapleton-St. George	11363	Queens	Bayside-Little Neck
10305	Staten Island	Stapleton-St. George	11364	Queens	Bayside-Little Neck
10306	Staten Island	South Beach-Tottenville	11365	Queens	Fresh Meadows
10307	Staten Island	South Beach-Tottenville	11366	Queens	Fresh Meadows
10308	Staten Island	South Beach-Tottenville	11367	Queens	Fresh Meadows
10309	Staten Island	South Beach-Tottenville	11368	Queens	West Queens
10310	Staten Island	Port Richmond	11369	Queens	West Queens
10312	Staten Island	South Beach-Tottenville	11370	Queens	West Queens
10314	Staten Island	Willowbrook	11372	Queens	West Queens
10451	Bronx	High Bridge-Morrisania	11373	Queens	West Queens
10452	Bronx	High Bridge-Morrisania	11374	Queens	Ridgewood-Forest Hills
10453	Bronx	Crotona-Tremont	11375	Queens	Ridgewood-Forest Hills
10454	Bronx	Hunts Point-Mott Haven	11377	Queens	West Queens
10455	Bronx	Hunts Point-Mott Haven	11378	Queens	West Queens
10456	Bronx	High Bridge-Morrisania	11379	Queens	Ridgewood-Forest Hills
10457	Bronx	Crotona-Tremont	11385	Queens	Ridgewood-Forest Hills
10458	Bronx	Fordham-Bronx Park	11411	Queens	Southeast Queens
10459	Bronx	Hunts Point-Mott Haven	11412	Queens	Jamaica
10460	Bronx	Crotona-Tremont	11413	Queens	Southeast Queens
10461	Bronx	Pelham-Throgs Neck	11414	Queens	Southwest Queens
10462	Bronx	Pelham-Throgs Neck	11415	Queens	Southwest Queens
10463	Bronx	Kingsbridge-Riverdale	11416	Queens	Southwest Queens
10464	Bronx	Pelham-Throgs Neck	11417	Queens	Southwest Queens
10465	Bronx	Pelham-Throgs Neck	11418	Queens	Southwest Queens
10466	Bronx	Northeast Bronx	11419	Queens	Southwest Queens
10467	Bronx	Fordham-Bronx Park	11420	Queens	Southwest Queens
10468	Bronx	Fordham-Bronx Park	11421	Queens	Southwest Queens
10469	Bronx	Northeast Bronx	11422	Queens	Southeast Queens
10470	Bronx	Northeast Bronx	11423	Queens	Jamaica
10471	Bronx	Kingsbridge-Riverdale	11426	Queens	Southeast Queens
10472	Bronx	Pelham-Throgs Neck	11427	Queens	Southeast Queens
10473	Bronx	Pelham-Throgs Neck	11428	Queens	Southeast Queens
10474	Bronx	Hunts Point-Mott Haven	11429	Queens	Southeast Queens
10475	Bronx	Northeast Bronx	11432	Queens	Jamaica
11004	Queens	Southeast Queens	11433	Queens	Jamaica
11005	Queens	Southeast Queens	11434	Queens	Jamaica
11101	Queens	Long Island City-Astoria	11435	Queens	Jamaica
11102	Queens	Long Island City-Astoria	11436	Queens	Jamaica
11103	Queens	Long Island City-Astoria	11691	Queens	Rockaway
11104	Queens	Long Island City-Astoria	11692	Queens	Rockaway
11105	Queens	Long Island City-Astoria	11693	Queens	Rockaway
11106	Queens	Long Island City-Astoria	11694	Queens	Rockaway
11201	Brooklyn	Downtown-Heights-Park Slope	11695	Queens	Rockaway
11203	Brooklyn	East Flatbush-Flatbush	11697	Queens	Rockaway

## References

[1] Wadhera R.K., Wadhera P., Gaba P., Figueroa J.F., Joynt Maddox K.E., Yeh R.W., Shen C. (2020). Variation in COVID-19 Hospitalizations and Deaths Across New York City Boroughs. JAMA.

[2] Zhang C.H., Schwartz G.G. (2020). Spatial Disparities in Coronavirus Incidence and Mortality in the United States: An Ecological Analysis as of May 2020. J. Rural Health.

[3] (2020). USA Facts. US Coronavirus Cases and Deaths. https://usafacts.org/visualizations/coronavirus-covid-19-spread-map/.

[4] U. S. Centers for Disease Control and Prevention (2020). COVID-19 in Racial and Ethnic Minority Groups.

[5] Yancy C.W. (2020). COVID-19 and African Americans. JAMA.

[6] Holtgrave D.R., Barranco M.A., Tesoriero J.M., Blog D.S., Rosenberg E.S. (2020). Assessing racial and ethnic disparities using a COVID-19 outcomes continuum for New York State. Ann. Epidemiol..

[7] Ogedegbe G., Ravenell J., Adhikari S., Butler M., Cook T., Francois F., Iturrate E., Jean-Louis G., Jones S.A., Onakomaiya D. (2020). Assessment of Racial/Ethnic Disparities in Hospitalization and Mortality in Patients with COVID-19 in New York City. JAMA Netw. Open.

[8] Benitez J., Courtemanche C., Yelowitz A. (2020). Racial and Ethnic Disparities in COVID-19: Evidence from Six Large Cities. J. Econ. Race Policy.

[9] Do D.P., Frank R. (2021). Unequal burdens: Assessing the determinants of elevated COVID-19 case and death rates in New York City’s racial/ethnic minority neighbourhoods. J. Epidemiol. Community Health.

[10] Kim B., Rundle A.G., Goodwin A.T.S., Morrison C.N., Branas C.C., El-Sadr W., Duncan D.T. (2021). COVID-19 testing, case, and death rates and spatial socio-demographics in New York City: An ecological analysis as of June 2020. Health Place.

[11] Li M., Yuan F. (2022). Historical Redlining and Resident Exposure to COVID-19: A Study of New York City. Race Soc. Probl..

[12] Oishi S., Cha Y., Schimmack U. (2021). The Social Ecology of COVID-19 Cases and Deaths in New York City: The Role of Walkability, Wealth, and Race. Soc. Psychol. Personal. Sci..

[13] Friedman S., Lee J.-W. (2021). COVID-19 mortality in New York City across neighborhoods by race, ethnicity, and nativity status. Geogr. Rev..

[14] Lieberman-Cribbin W., Tuminello S., Flores R.M., Taioli E. (2020). Disparities in COVID-19 Testing and Positivity in New York City. Am. J. Prev. Med..

[15] Moreland A., Gillezeau C., Eugene A., Alpert N., Taioli E. (2022). Ecologic study of influenza vaccination uptake and COVID-19 death rate in New York City. BMC Public Health.

[16] Li R., Huang Y. (2023). COVID-19 pandemic and minority health disparities in New York City: A spatial and temporal perspective. Environ. Plan. B Urban Anal. City Sci..

[17] Huang Y., Li R. (2022). The lockdown, mobility, and spatial health disparities in COVID-19 pandemic: A case study of New York City. Cities.

[18] Bilal U., Tabb L.P., Barber S., Diez Roux A.V. (2021). Spatial Inequities in COVID-19 Testing, Positivity, Confirmed Cases, and Mortality in 3 U.S. Cities: An Ecological Study. Ann. Intern. Med..

[19] Lieberman-Cribbin W., Alpert N., Flores R., Taioli E. (2021). A risk index for COVID-19 severity is associated with COVID-19 mortality in New York City. BMC Public Health.

[20] NYU Furman Center (2018). State of New York City’s Housing and Neighborhoods in 2018.

[21] New York City Department of Health and Mental Hygiene (2021). COVID-19 Cases by Zip Code in NYC. https://github.com/nychealth/coronavirus-data.

[22] IPUMS NHGIS. https://www.nhgis.org/.

[23] New York City Department of Health and Mental Hygiene Community Health Survey 2016–2018 Restricted Use Dataset. https://nycdohmh.surveymonkey.com/r/EpiDataForm.

[24] U. S. Census Bureau (2017). American Community Survey 2014 Questionnaire Booklet.

[25] U. S. Census Bureau Table B03002. Hispanic or Latino Origin by Race. 2014–2018: American Community Survey 5-Year Estimates Detailed Tables.

[26] Sampson R.J., Raudenbush S.W., Earls F. (1997). Neighborhoods and violent crime: A multilevel study of collective efficacy. Science.

[27] Glaeser E.L., Gorback C., Redding S.J. (2022). JUE Insight: How much does COVID-19 increase with mobility? Evidence from New York and four other U.S. cities. J. Urban Econ..

[28] Hanigan I., Hall G., Dear K.B. (2006). A comparison of methods for calculating population exposure estimates of daily weather for health research. Int. J. Health Geogr..

[29] Martin D. (1989). Mapping population data from zone centroid locations. Trans. Inst. Br. Geogr..

[30] Richardson S., Hirsch J.S., Narasimhan M., Crawford J.M., McGinn T., Davidson K.W., Barnaby D.P., Becker L.B., Chelico J.D. (2020). Presenting Characteristics, Comorbidities, and Outcomes among 5700 Patients Hospitalized with COVID-19 in the New York City Area. JAMA.

[31] Chilimuri S., Sun H., Alemam A., Manthri N., Shehi E., Tejada J., Yugay A., Nayudu S. (2020). Predictors of Mortality in Adults Admitted with COVID-19: Retrospective Cohort Study from New York City. West. J. Emerg. Med..

[32] Arasteh K. (2021). Prevalence of Comorbidities and Risks Associated with COVID-19 Among Black and Hispanic Populations in New York City: An Examination of the 2018 New York City Community Health Survey. J. Racial Ethn. Health Disparities.

[33] Corona G., Pizzocaro A., Vena W., Rastrelli G., Semeraro F., Isidori A.M., Pivonello R., Salonia A., Sforza A., Maggi M. (2021). Diabetes is most important cause for mortality in COVID-19 hospitalized patients: Systematic review and meta-analysis. Rev. Endocr. Metab. Disord..

[34] Kastora S., Patel M., Carter B., Delibegovic M., Myint P.K. (2022). Impact of diabetes on COVID-19 mortality and hospital outcomes from a global perspective: An umbrella systematic review and meta-analysis. Endocrinol. Diabetes Metab..

[35] Strully K., Yang T.-C., Liu H. (2021). Regional variation in COVID-19 disparities: Connections with immigrant and Latinx communities in U.S. counties. Ann. Epidemiol..

[36] Woolf S.H., Chapman D.A., Lee J.H. (2021). COVID-19 as the Leading Cause of Death in the United States. JAMA.

[37] U. S. Census Bureau (2020). Table B05003D. Sex by Age by Nativity and Citizenship Status (Asian Alone). 2019: American Community Survey 5-Year Estimates Detailed Tables. https://data.census.gov.

[38] Phelan J.C., Link B.G. (2015). Is Racism a Fundamental Cause of Inequalities in Health?. Annu. Rev. Sociol..

[39] Williams D.R., Collins C. (2001). Racial residential segregation: A fundamental cause of racial disparities in health. Public Health Rep..

[40] Massey D., Denton N.A. (1993). American Apartheid: Segregation and the Making of the Underclass.

[41] Massey D.S., Tannen J. (2015). A Research Note on Trends in Black Hypersegregation. Demography.

[42] Hotchkiss M. (2015). Hypersegregated Cities Face Tough Road to Change. https://www.princeton.edu/news/2015/05/18/hypersegregated-cities-face-tough-road-change.

[43] Feldmeyer B., Madero-Heranandez A., Rojas-Gaona C.E., Sabon L.C. (2019). Immigration, Collective Efficacy, Social Ties, and Violence: Unpacking the Mediating Mechanisms in Immigration Effects on Neighborhood-Level Violence. Race Justice.

[44] Logan J.R., Stults B. (2011). Diversity and Disparities: Residential Segregation by Race/Ethnicity–Sortable Lists of Segregation by Race/Ethnicity for the 200 Largest Cities. https://s4.ad.brown.edu/projects/diversity/segregation2020/Default.aspx.

[45] Torrats-Espinosa G. (2021). Using machine learning to estimate the effect of racial segregation on COVID-19 mortality in the United States. Proc. Natl. Acad. Sci. USA.

